# Supplementary material for: Inhibiting SARS-CoV-2 infection in vitro by suppressing its receptor, angiotensin-converting enzyme 2, via aryl-hydrocarbon receptor signal
Source: Sci Rep. 2021 Aug 17;11:16629. doi: 10.1038/s41598-021-96109-w (PMC8371152; doi:10.1038/s41598-021-96109-w)
Supplement: Supplementary file 1 — Supplementary Information. [file 41598_2021_96109_MOESM1_ESM.pdf]

Inhibiting SARS-CoV-2 infection *in vitro* by suppressing its receptor, angiotensin-converting enzyme 2, via aryl-hydrocarbon receptor signal

Keiji Tanimoto[1,*], Kiichi Hirota[2], Takahiro Fukazawa[3], Yoshiyuki Matsuo[2], Toshihito Nomura[4], Nazmul Tanuza[4], Nobuyuki Hirohashi[1], Hidemasa Bono[5], and Takemasa Sakaguchi[4]

[1]Department of Radiation Disaster Medicine, Research Institute for Radiation Biology and Medicine, Hiroshima University, Hiroshima 734-8553, Japan

[2]Department of Human Stress Response Science, Institute of Biomedical Science, Kansai Medical University, Hirakata 573-1010, Japan

[3]Natural Science Center for Basic Research and Development, Hiroshima University, Hiroshima 734-8553, Japan

[4]Department of Virology, Graduate School of Biomedical and Health Sciences, Hiroshima University, Hiroshima 734-8553, Japan

[5]Program of Biomedical Science, Graduate School of Integrated Sciences for Life, Hiroshima University, Higashi-Hiroshima, 739-0046, Japan

* To whom correspondence should be addressed. Tel: +81 (0)82 257 5841; Fax: +81 (0)82 256 7105; Email: ktanimo@hiroshima-u.ac.jp

Supplementary Information

Supplementary Fig. 1S. Expression levels of *ACE2* gene in various human cell lines.

(a) Expression levels of *ACE2* genes in various cell lines were evaluated by qRT-PCR. Relative gene expression levels were calculated by using *ACTB* expression as the denominator for each cell line (n = 3). For all quantitative values, the average and SD are shown. (b, c) Expression levels of *CYP1A1* and *ACE2* genes in various concentrations of CSE treated PC9 (lung origin) and HSC2 (oral cavity origin) cells for 24 hours were evaluated by qRT-PCR. Relative gene expression levels were calculated by using *ACTB* expression as the denominator for each cell line (n = 3). For all quantitative values, the average and SD are shown. Statistical significance was calculated for the indicated paired samples with ** representing $P < 0.01$.

Supplementary Fig. 2S. RNA-seq analyses of HepG2 cells treated with AHR agonists.

Venn diagrams of genes upregulated (a) and downregulated (e) with FICZ or OMP treatment in HepG2 cells are shown. Upregulated (b, c) and downregulated (f, g) genes commonly regulated were enriched by GO term and TRRUST. AHR and ARNT are indicated with red boxes. Enrichment analyses of upregulated (d) and downregulated (h) genes were also performed with Coronascape (coronascape.org), a one-stop meta-analysis resource of large-

scale omics data. (i) Gene expression levels (read counts) of *TMPRSS2* (left), *FURIN* (middle), and *CTSL* (cathepsin L) (right) genes in HepG2 analyzed with RNA-seq are shown.

Supplementary Fig. 3S. Effects of tryptophan metabolites and proton pump inhibitors on *ACE2* suppression.

Expression levels of *CYP1A1* (upper), and *ACE2* (lower) genes in various concentrations of tryptophan metabolites (a) or proton pump inhibitors (b) treated HepG2 cells were evaluated by quantitative RT-PCR. Relative gene expression levels were calculated by using *ACTB* expression as the denominator for each cell line (n = 3). For all quantitative values, the average and SD are shown. Statistical significance was calculated for the indicated paired samples with * representing $P < 0.05$ and ** $P < 0.01$.

Supplemental Table 1S. Primer and probe sequences for qRT-PCR.

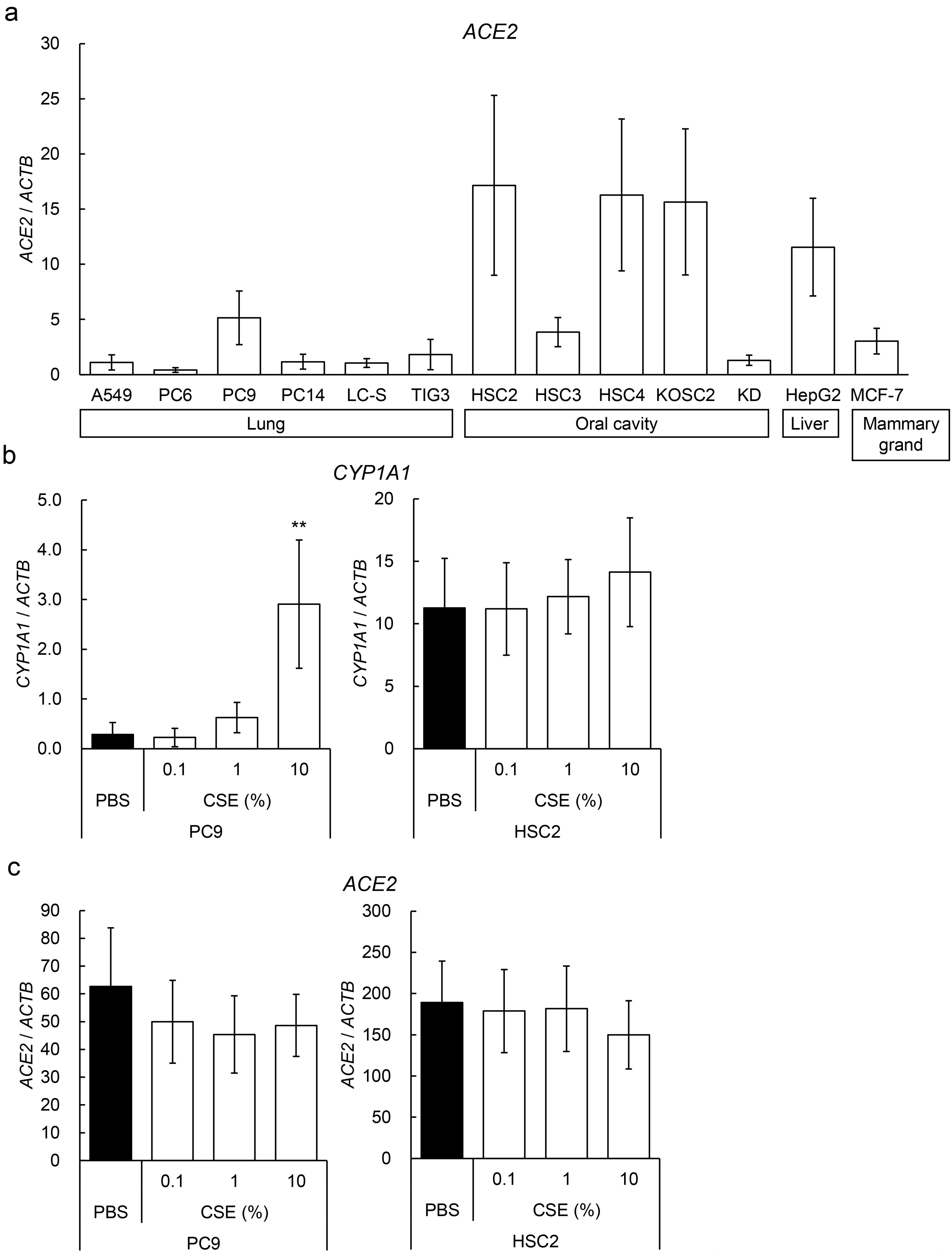

Supplementary Fig.1S

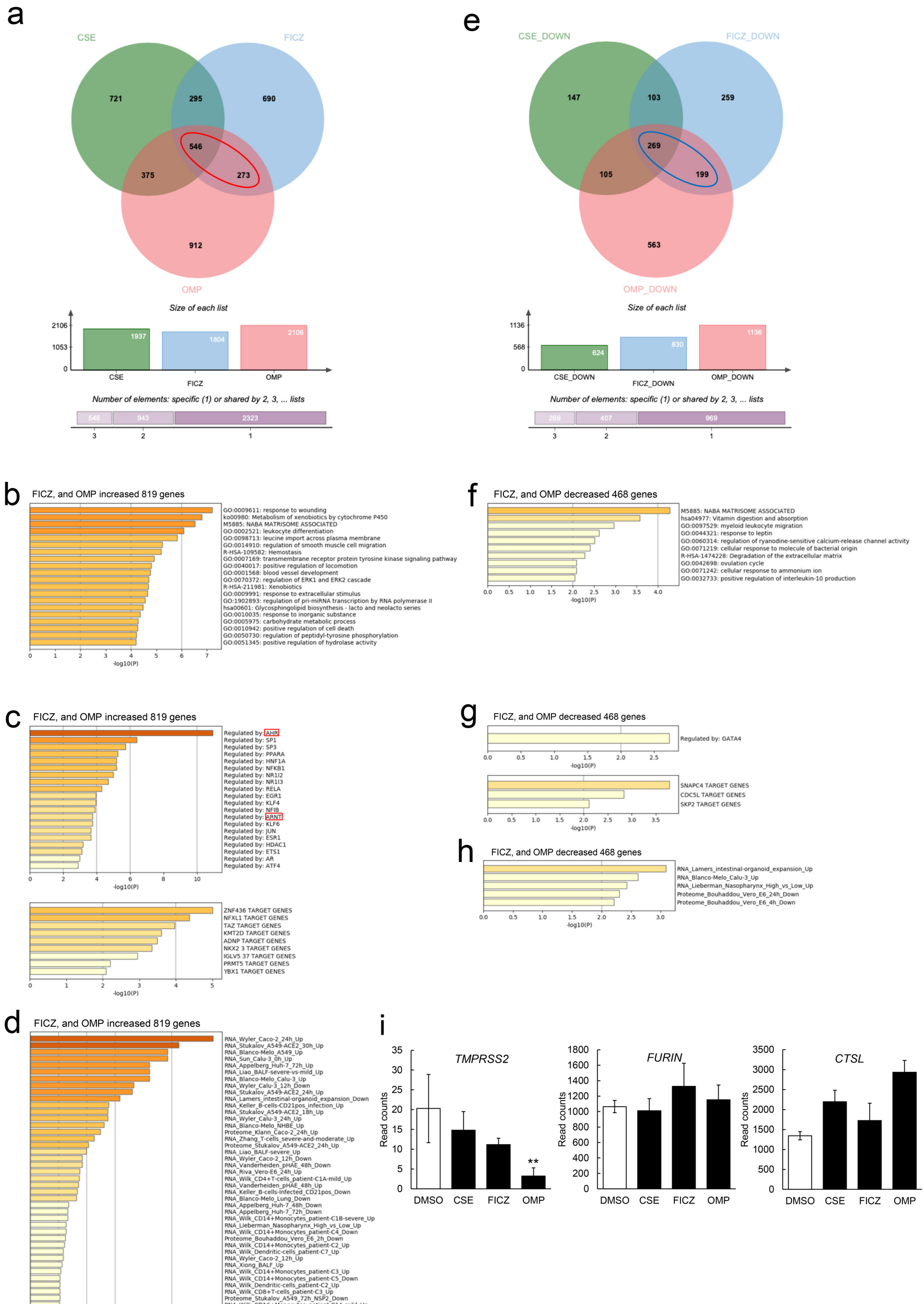

Supplementary Fig.2S

a

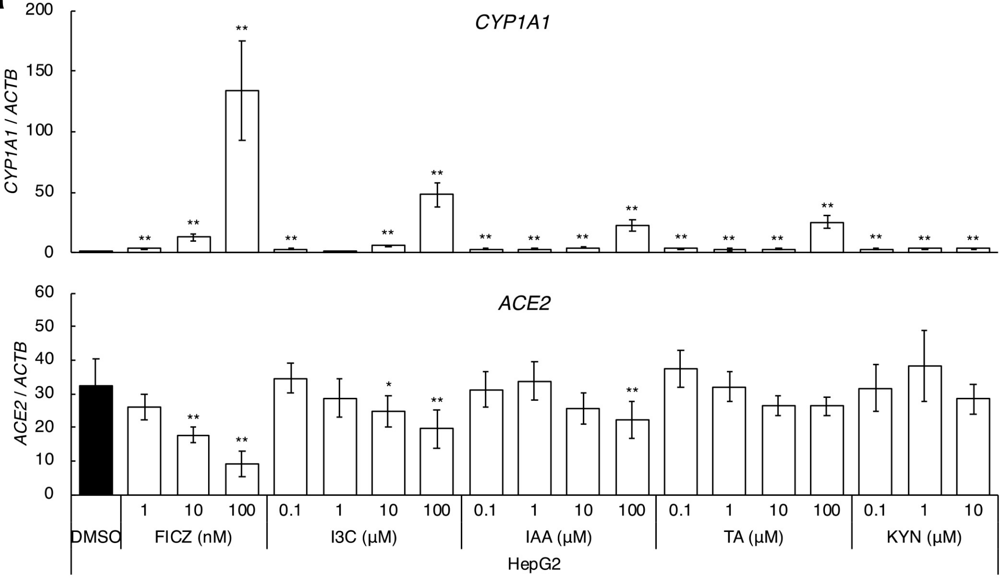

b

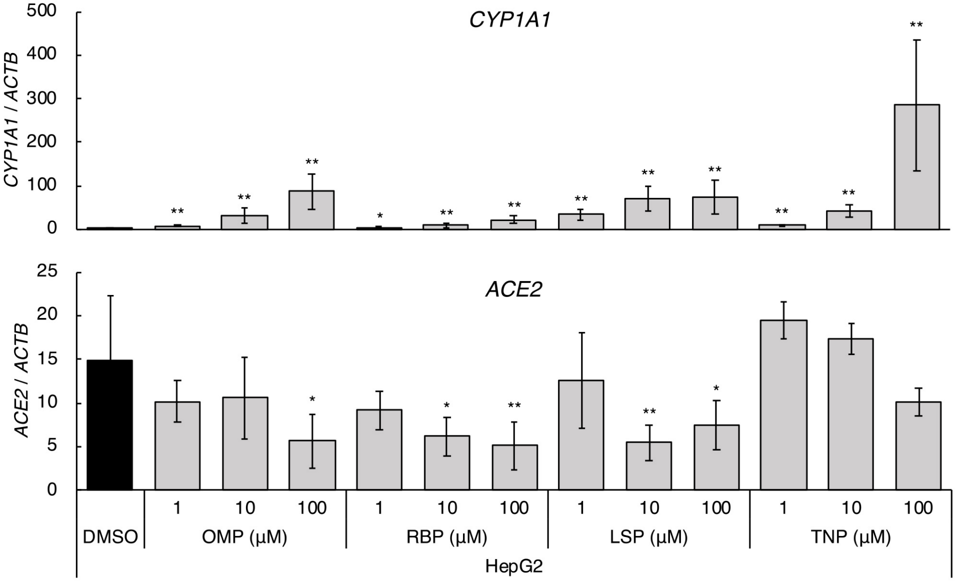

Supplementary Fig.3S

Supplementary Table 1S

Primer sets and MGB probes for quantitative RT-PCR.

Human *CYP1A1*_F: 5’- ACCTTCCCTGATCCTTGTGA -3’

Human *CYP1A1*_R: 5’- GGAGATTGGGAAAAGCATGA -3’

Human *CYP1A1*_Probe: UPL #10 (Roche)

Human *ACE2*_F: 5’- TTCTGTCACCCGATTTTCAA -3’

Human *ACE2*_R: 5’- TCCCAACAATCGTGAGTGC -3’

Human *ACE2*_Probe: UPL #4 (Roche)

Human *AHR*_F: 5’- CAACATCACCTACGCCAGTC -3’

Human *AHR*_R: 5’- GCTTGGAAGGATTTGACTTGA -3’

Human *AHR*_Probe: UPL #33 (Roche)

Primate *ACTB2*_F: 5’- CACCATGTACCCTGGCATC -3’

Primate *ACTB*_R: 5’- ACGGAGTACTTGCGCTCAG -3’

Primate *ACTB*_Probe: UPL #37 (Roche)

Primate *ACE2*_F: 5’- AGTCTGCCATCCCACAGC -3’

Primate *ACE2*_R: 5’- CGTCCATTGTCACCTTTGTG -3’

Primate *ACE2*_Probe: UPL #60 (Roche)
